# OBSTRUCTIVE SLEEP APNEA RISK AND LONGITUDINAL TRAJECTORY OF FUNCTIONAL HEARING LOSS OVER EIGHT YEARS OF FOLLOW-UP

**DOI:** 10.1093/geroni/igad104.1618

**Published:** 2023-12-21

**Authors:** Kening Jiang, Adam Spira, Nicholas Reed, Frank Lin, Jennifer Deal

**Affiliations:** Johns Hopkins Bloomberg School of Public Health, Baltimore, Maryland, United States; Johns Hopkins Bloomberg School of Public Health, Baltimore, Maryland, United States; Johns Hopkins Bloomberg School of Public Health, Baltimore, Maryland, United States; Johns Hopkins, Baltimore, Maryland, United States; Johns Hopkins University, Baltimore, Maryland, United States

## Abstract

Hearing loss prevention is important for physical, cognitive and psychosocial health among older adults. Obstructive sleep apnea (OSA) might cause functional hearing loss through ischemic damages to the cochlea or central auditory processing, but there is a lack of longitudinal evidence. The National Health and Aging Trends Study (NHATS) is a longitudinal panel survey of Medicare beneficiaries aged 65+ years. STOP-Bang is a validated screening tool for OSA risk and a subsample of NHATS round 3 participants responded to a modified version (age>50, male, high blood pressure, body mass index >35 kg/m2, snore loudly, observed apnea, tiredness; range 0-7, elevated risk if ≥3). Functional hearing loss at follow-up (rounds 3-11) was defined as self-reported deafness, hearing aid use, or inability to hear well enough to use the telephone or have conversations in a room with the television or radio on. We used generalized estimating equations logistic regression with inverse probability attrition weighting, adjusting for demographics and comorbidities. Among 1,433 participants, elevated OSA (58%) was associated with higher odds of hearing loss at baseline (odds ratio:1.50, 95% confidence interval[CI]:1.08, 2.10). Both OSA groups had significant annual increases in odds of functional hearing loss (Low:1.07, 95% CI:1.01, 1.13; Elevated:1.12, 95% CI:1.07, 1.18) during the first 4 years. Afterwards, the low OSA risk group continued to have significant increase in odds of functional hearing loss but not the elevated OSA risk group. OSA might be a novel risk factor for hearing loss. Screening and treating OSA might be important for hearing loss prevention.

